# Facilitating Out-of-Home Caregiving Through Health Information Technology: Survey of Informal Caregivers’ Current Practices, Interests, and Perceived Barriers

**DOI:** 10.2196/jmir.2472

**Published:** 2013-07-10

**Authors:** Donna M Zulman, John D Piette, Emily C Jenchura, Steven M Asch, Ann-Marie Rosland

**Affiliations:** ^1^Center for Health Care EvaluationVA Palo Alto Health Care SystemMenlo Park, CAUnited States; ^2^Division of General Medical DisciplinesStanford UniversityStanford, CAUnited States; ^3^Center for Clinical Management ResearchVA Ann ArborAnn Arbor, MIUnited States; ^4^Division of General Internal MedicineUniversity of Michigan Medical SchoolAnn Arbor, MIUnited States

**Keywords:** caregivers, chronic disease, medical informatics

## Abstract

**Background:**

Many patients with chronic conditions are supported by out-of-home informal caregivers—family members, friends, and other individuals who provide care and support without pay—who, if armed with effective consumer health information technology, could inexpensively facilitate their care.

**Objective:**

We sought to understand caregivers’ use of, interest in, and perceived barriers to health information technology for out-of-home caregiving.

**Methods:**

We conducted 2 sequential Web-based surveys with a national sample of individuals who provide out-of-home caregiving to an adult family member or friend with a chronic illness. We queried respondents about their use of health information technology for out-of-home caregiving and used multivariable regression to investigate caregiver and care-recipient characteristics associated with caregivers’ technology use for caregiving.

**Results:**

Among 316 out-of-home caregiver respondents, 34.5% (109/316) reported using health information technology for caregiving activities. The likelihood of a caregiver using technology increased significantly with intensity of caregiving (as measured by number of out-of-home caregiving activities). Compared with very low intensity caregivers, the adjusted odds ratio (OR) of technology use was 1.88 (95% CI 1.01-3.50) for low intensity caregivers, 2.39 (95% CI 1.11-5.15) for moderate intensity caregivers, and 3.70 (95% CI 1.62-8.45) for high intensity caregivers. Over 70% (149/207) of technology nonusers reported interest in using technology in the future to support caregiving. The most commonly cited barriers to technology use for caregiving were health system privacy rules that restrict access to care-recipients’ health information and lack of familiarity with programs or websites that facilitate out-of-home caregiving.

**Conclusions:**

Health information technology use for out-of-home caregiving is common, especially among individuals who provide more intense caregiving. Health care systems can address the mismatch between caregivers’ interest in and use of technology by modifying privacy policies that impede information exchange.

## Introduction

As American society becomes increasingly mobile and households decline in size [1], many chronically ill patients find themselves without a caregiver in their home. By some accounts, as many as 70% of the estimated 66 million Americans who provide unpaid assistance to ill or older adults live apart from their care recipient [2,3]. Out-of-home caregiving is especially common when the care recipient is an elderly relative [4], and this trend is likely to become more pronounced as the population ages [5].

Out-of-home caregivers face a number of unique challenges. Some caregivers experience emotional stress, guilt, and helplessness related to living apart from loved ones who are in need of care [6-8]. Additional challenges may arise when caregivers live at an increased distance from their care recipients [3,9]. In one assessment, costs associated with out-of-home caregiving doubled as travel increased from 1 to 3 hours (US $386 per month) to more than 3 hours (US $674 per month). Long-distance caregivers reported spending 22 hours per month on average assisting their care recipients with instrumental activities, such as transportation, shopping, and finances, and more than half of them visited their care recipient at least a few times per month, despite a mean travel distance of 450 miles [9].

Health information technology may provide an opportunity to support out-of-home caregivers’ activities. Consumer health information technology encompasses a wide range of technologies that allow patients to participate in their health care via electronic means [10]. Examples of health information technology include electronic personal health records, applications that facilitate chronic condition management (eg, programs for tracking blood pressure and glucose), Internet resources with medication and disease information, and tools that facilitate communication with health care providers. Many of these applications may be of value to out-of-home caregivers as well, for example by alleviating uncertainty about a care recipient’s symptoms and status, or enhancing information exchange with a care recipient’s health care team.

Despite recent discussions that health information technology could facilitate caregiving from afar [4], there have been few assessments of current and potential technology use for this purpose. We conducted a survey of individuals who care for an out-of-home adult family member or friend with a chronic condition. Our objectives were to (1) determine rates of, and interest in, health information technology use for out-of-home caregiving activities, (2) examine caregiver and patient characteristics associated with technology use, and (3) identify barriers to out-of-home caregivers’ use of technology that may be overcome through enhanced technology and associated policies.

## Methods

### Survey Design and Administration

This paper reports findings from 2 sequential surveys of individuals who support family members and friends with chronic illness. The surveys for this study were administered by Knowledge Networks, a research firm that maintains a large, nationally representative survey panel of adults. Knowledge Networks’ panel is very similar to the United States population with respect to race/ethnicity, gender, educational attainment, and income [11]. In return for their participation in the panel, members receive Internet access and computing equipment at no cost [12,13].

For this study, we identified potential participants using data from a previous study (Wave 1: January 26 to February 16, 2010) [14]. We identified 748 individuals from the Wave 1 cohort who (1) had an adult family member or friend with a chronic illness (including diabetes, chronic heart disease, chronic lung disease, arthritis, and/or depression), (2) lived apart from this person for more than half of the year, and (3) reported a high willingness to help this person with his or her health. Of note, individuals whose care recipients were living in a long-term care facility or required assistance with basic activities of daily living were excluded from the Wave 1 cohort because the focus of this earlier study was on support for independent and ambulatory adults with a chronic illness [14].

**Figure 1:**
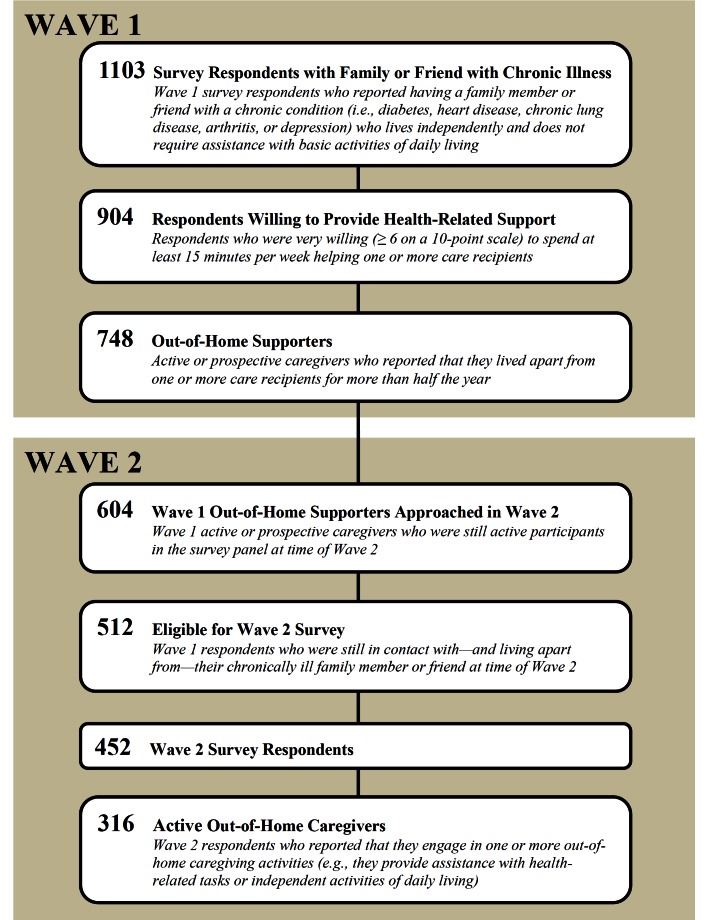
Wave 1 and Wave 2 survey populations.

For the current study (Wave 2: January 20 to February 21, 2011), we invited the 604 Wave 1 participants who were still active Knowledge Networks panelists to complete a follow-up survey about their use of health information technology to support their out-of-home care recipient (Multimedia Appendix 1). Of the 512 individuals who completed a screening questionnaire (response rate 84.8%), 452 reported that they were still in touch with—and living apart from—the care recipient whom they had identified in Wave 1. In this paper, we report survey findings for the subgroup of 316 respondents who we identified as active out-of-home caregivers (ie, they reported engaging in 1 or more out-of-home caregiving activities, described subsequently, to support their care recipient) (Figure 1). Specifically, we report these respondents’ use of, interest in, and barriers to health information technology for out-of-home caregiving activities, and we describe caregiver and patient characteristics associated with technology use.

### Survey Measures

#### Dependent Variable: Use of Health Information Technology for Out-of-Home Caregiving

Our dependent variable was use of health information technology for out-of-home caregiving activities. We asked Wave 2 survey respondents, “In the past year, in what ways have you used the computer, Internet, or email to help [your care recipient] manage his or her health.” Textbox 1 indicates the response options provided to respondents. We dichotomized respondents based on whether they reported any versus no use of 1 or more of these technologies for caregiving.

**Survey questions regarding use of health information technology for caregiving.**

In the past year, in what ways have you used the computer, Internet, or email to help [your care recipient] manage his or her health:

1. I helped him or her find health information online
2. I sent messages to his or her doctor or other health care provider by email
3. I helped him or her track his or her health information (for example, their blood pressure, blood sugar, or medication use) on a computer
4. I helped him or her access his or her health records through a system linked to his or her health care provider (ie, a personal health record system or health portal)
5. I helped him or her use a health portal or personal health record system that is available through his or her health care provider
6. I helped him or her fill medications or medical supplies online
7. I helped him or her look up medical test results online
8. Other ways: _____
9. I have not used the computer, Internet, or email for any of the above

#### Independent Variables: Caregiver and Care Recipient Characteristics

We measured out-of-home caregiving intensity by assessing respondents’ involvement in health-related activities that might be amenable to out-of-home assistance. These included assistance with independent activities of daily living in the past 3 months, assistance with health-related tasks in the past 3 months, discussions about health with the care recipient usually or always in conversations, communication with the care recipient’s physician in the past year, and guidance given to the care recipient about questions to ask a health care provider in the past year. After examining the distribution of respondents’ participation in these activities, we generated an out-of-home caregiving intensity index comprising the sum of these items (1 = very low, 2 = low, 3 = moderate, 4-5 = high).

We queried caregivers about their comfort with technology (including computers, the Internet, email, text messaging, and learning how to use new programs on a computer or the Internet), using survey questions that were previously piloted in an evaluation of a Web-based caregiving intervention [15]. Factor analysis demonstrated that all of the questions loaded onto a single factor (Cronbach alpha=.89); thus, the 5 items were combined into a single measure comprising the sum of technology modalities or tasks with which respondents agreed or strongly agreed they felt comfortable (0 = very low, 1-2 = low, 3-4 = moderate, 5 = high).

We obtained additional information about caregiver characteristics from the Wave 1 survey and from the Knowledge Networks database of panel members, including caregivers’ age, sex, race/ethnicity, household income, education, health status, and whether the respondent had Internet access prior to enrolling in Knowledge Networks.

Finally, we obtained caregiver-reported information about care recipients’ characteristics. In Wave 1, we asked caregivers to rank their care recipient’s health status (5-point scale, poor to excellent) [16] and to report whether their care recipient had a hospital admission or emergency room visit in the past year. In addition, we constructed a single variable, unmet health or health care needs, based on whether the care recipient sometimes to frequently discussed any of the following issues with the caregiver over the past 6 months: pain or bothersome symptoms, medication side effects, confusion about a doctor’s advice, unanswered questions that were asked of the doctor, or insufficient support to manage his or her health problems. We also collected information about the care recipient’s relationship with the caregiver and their geographic distance from one another (Wave 1), and about the care recipient’s age and whether he or she uses the Internet (Wave 2).

#### Additional Descriptive Variables

If respondents indicated that they did not have experience using technology for a specific caregiving purpose, we asked about their interest in using technology for that purpose in the future if it would help their care recipients improve their health. We also queried all respondents about barriers to technology use for out-of-home caregiving, including insufficient time, unfamiliarity with relevant programs or websites, health problems, and privacy rules that restrict access to their care recipients’ health information.

### Data Analysis

We first examined rates of health information technology use and interest in technology for specific caregiving activities. We then used multivariable logistic regression models to examine the association between out-of-home caregiving intensity and a respondent’s use of technology for caregiving activities, adjusting for comfort with technology, as well as caregiver’s age, sex, race/ethnicity, education, income, and health status. We constructed a similar model to investigate whether specific care recipient characteristics were associated with a caregiver’s use of technology. For this model, we included the care recipient’s age, health status, incidence of hospitalizations, and incidence of emergency room visits over the previous year, the presence of unmet health or health care needs, the care recipient’s geographic distance from the caregiver, and whether the care recipient uses the Internet. Finally, we examined common barriers cited by technology-using and technology-nonusing caregivers that prevent them from using technology (or using it more frequently) for caregiving activities.

We used Stata 12.0 (StataCorp LP, College Station, TX, USA) to perform all analyses. Rates of item-level missing data were less than 8% for all covariates used in analyses. Regression diagnostic procedures yielded no evidence of multicollinearity in any of the regression models. Datasets were deidentified before receipt from Knowledge Networks. Both waves of the study were classified as exempt by the Institutional Review Board at the University of Michigan.

## Results

Characteristics of the 316 survey respondents and their care recipients are described in Table 1. There were 109 (34.5%) out-of-home caregivers who reported using health information technology for caregiving activities, 24 (26.1%) of whom reported a frequency of monthly or more. Among these technology users, the most common purpose for technology use was to help a care recipient find health information online (70.6%), whereas sending emails to health care providers, tracking health information, accessing health records, filling medications, and looking at medical test results online were each cited by fewer than 15% of technology users.

Among the 207 respondents who reported no use of technology for caregiving, 122 (58.9%) stated that the reason for this was that their care recipient did not need their help in this way. However, 150 (73.0%) expressed a willingness to use technology in the future if it would help their care recipient with his or her health, for example 139 (67.8%) to find health information, 111 (53.6%) to track personal health information, and 104 (50.2%) to fill medications or medical supplies (Table 2). In addition, 90 of the 109 (83.0%) active technology users were interested in expanding their technology use in the future to support at least 1 additional caregiving task that they were not already engaging in using technology. Of note, active technology users were interested in expanding their technology use to interact with their care recipients’ health care system, for example to communicate with health care providers (57/101, 56.4%) and help their care recipients look up medical test results online (68/102, 66.7%).

Multivariable logistic regression revealed that greater out-of-home caregiving intensity was significantly associated with caregivers’ likelihood of using health information technology. Compared to respondents with very low intensity caregiving roles, the adjusted odds of caregiving-related technology use increased steadily when caregiving intensity was low (adjusted odds ratio [OR] 1.88, 95% CI 1.01-3.50, *P*=.05), moderate (adjusted OR 2.39, 95% CI 1.11-5.15, *P*=.03), and high (adjusted OR 3.70, 95% CI 1.62-8.45, *P*=.002) (Table 3). The likelihood of technology use also increased markedly with a caregiver’s comfort using technology. None of the other caregiver characteristics that we assessed were associated with technology use (Multimedia Appendix 2).

In a separate multivariable logistic regression analysis investigating whether care recipient characteristics (including age, geographic distance from the caregiver, and health status) were associated with a respondent’s use of technology for caregiving, no significant relationships were observed (Multimedia Appendix 3).

Nearly half (49.4%, 156/316) of all respondents, 40.1% (83/207) of technology nonusers, and 67.0% (73/109) of technology users reported that there were barriers to their use of technology for out-of-home caregiving. Among the respondents reporting barriers to technology use, 57.7% (28.5% of all respondents) cited privacy rules of their care recipient’s health care provider, and 58.3% (28.8% of all respondents) cited unfamiliarity with programs or websites that facilitate out-of-home caregiving. In contrast, very few respondents reported that insufficient time, computer/Internet complexity, distrust in the Internet, or their own health limitations impeded their use of technology for caregiving (Table 4). There were few differences in the frequency of barriers cited by technology users and technology nonusers, with the exception that active technology users were more than twice as likely as nonusers to report that privacy rules impeded their use of technology for caregiving (53/109, 48.6% vs 37/207, 17.9%, respectively).

**Table 1:** Description of study population (N=316).^a^

Characteristics			n (%)

**Out-of-home caregivers**			
	**Age**		
		18-29	38 (12.0)
		30-44	86 (27.2)
		45-59	101 (32.0)
		≥60	91 (28.8)
	Female		199 (63.0)
	**Education**		
		Less than high school	29 (9.2)
		High school degree	72 (22.8)
		Some college	112 (35.4)
		College degree or higher	103 (32.6)
	**Race/ethnicity**		
		White, Non-Hispanic	189 (59.8)
		Black, Non-Hispanic	71 (22.5)
		Hispanic	56 (17.7)
	**Geographic Region**		
		Northwest	57 (18.0)
		Midwest	62 (19.6)
		South	130 (41.1)
		West	67 (21.2)
	**Out-of-home caregiving activities (time frame)**		
		Assistance with independent activities of daily living (past 3 months) (N=314)	138 (44.0)
		Assistance with health-related tasks (past 3 months) (N=312)	69 (22.1)
		Frequent discussions about health with care recipient (N=316)	131 (41.5)
		Phone conversations with care recipient’s doctor (past 12 months) (N=310)	43 (13.9)
		Suggested questions for care recipient to ask health care provider (past 12 months) (N=308)	262 (85.1)
	Independent Internet access^b^		241 (76.3)
	**Comfort with technology**		
		Very low/low	88 (27.9)
		Moderate	114 (36.1)
		High	114 (36.1)

**Care recipients** ^c^			
	**Age (N=313)**		
		<50	68 (21.7)
		60-64	90 (28.8)
		65-74	83 (26.5)
		≥75	72 (23.0)
	Use Internet		183 (57.9)
	**Health status**		
		Very good or excellent	53 (16.8)
		Good	121 (38.3)
		Fair or poor	142 (44.9)
	**Relationship with caregiver**		
		Spouse/partner	4 (1.3)
		Adult child	26 (8.2)
		Sibling	88 (27.9)
		Parent or parent-in-law	124 (39.2)
		Other relative/friend	74 (23.4)
	**Distance from caregiver (N=312)**		
		<5 miles	74 (23.7)
		5-20	85 (27.2)
		21-100	44 (14.1)
		>100	109 (34.9)

^a^N=316 unless otherwise specified.

^b^Knowledge Networks provides Internet access to panel participants who do not have independent access.

^c^All care recipient characteristics are caregiver-reported.

**Table 2:** Health information technology functions that are of interest to out-of-home caregivers for adults with chronic conditions.

Technology function	Current technology nonusers, % (proportion of respondents)	Current technology users^a^, % (proportion of respondents)
Help care recipient find health information online	67.8 (139/205)	80.7 (25/31)
Help care recipient track his or her health information (eg, blood pressure, blood sugar, or medications)	53.6 (111/207)	61.6 (61/99)
Help care recipient look up medical test results online	52.2 (108/207)	66.7 (68/102)
Help care recipient use a health portal or personal health record system	51.2 (106/207)	56.7 (59/104)
Help care recipient fill medications or medical supplies online	50.2 (104/207)	54.3 (51/94)
Help care recipient keep track of his or her health records on the computer	49.3 (101/205)	63.1 (65/103)
Send email messages to care recipient’s doctor or other health care provider	44.9 (92/205)	56.4 (57/101)
Interest in one or more of the above functions	73.0 (150/207)	83.0 (90/109)

^a^Respondents who reported current technology use for one or more caregiving tasks were asked about their interest in expanding their use of technology for additional caregiving tasks in the future if it would help their care recipient manage his or her health.

**Table 3:** Out-of-home caregivers’ characteristics associated with their use of health information technology to support individuals with chronic conditions^a^ (N=316; 301 of whom are in multivariate model).

Caregiver characteristics		n (%)	Unadjusted OR (95% CI)	Adjusted OR (95% CI)
**Out-of-home caregiving intensity** ^b^				
	Very low	125 (39.6)		
	Low	104 (32.9)	1.82 (1.03-3.23)	1.88 (1.01-3.50)
	Moderate	49 (15.5)	2.18 (1.08-4.41)	2.39 (1.11-5.15)
	High	38 (12.0)	3.91 (1.83-8.36)	3.70 (1.62-8.45)
**Caregiver’s comfort with technology** ^b^				
	Very low	46 (14.6)		
	Low	42 (13.3)	1.64 (0.61-4.42)	1.23 (0.41-3.67)
	Moderate	114 (36.1)	2.31 (1.01-5.26)	2.09 (0.87-5.02)
	High	114 (36.1)	2.88 (1.27-6.54)	3.49 (1.34-9.11)

^a^Multivariable logistic regression model adjusted for caregiver’s age, education, income, race/ethnicity, and health status (see Multimedia Appendix 2 for results from full model).

^b^Categories described in detail in Multimedia Appendix 2. In the presented analysis, caregiving intensity was analyzed as categorical indicator variables. When caregiving intensity was analyzed as a continuous variable in a secondary analysis, the relationship with technology use had an adjusted OR of 1.54 (95% CI 1.20-1.98, *P*=.001).

**Table 4:** Barriers to health information technology use for out-of-home caregiving.^a^

Barriers	Current technology nonusers, % (n=207)	Current technology users, % (n=109)
Unfamiliarity with programs or websites that facilitate out-of-home caregiving	29.5	27.5
Privacy rules of care recipient’s health care provider	17.9	48.6
Insufficient time	7.3	12.8
Computer/Internet too complicated	8.2	5.5
Distrust in Internet for health-related information	6.8	3.7
Health or functional limitations	1.5	1.8
One or more of the above barriers	40.1	67.0

^a^Health information technology nonusers and users were asked to indicate all of the barriers that impede their use of technology or their more frequent use of technology, respectively, to help their care recipients with their health.

## Discussion

### Principal Findings

In this national survey of out-of-home caregivers for a chronically ill family member or friend, more than one-third (34.5%, 109/316) reported using health information technology to facilitate caregiving activities, and technology use was significantly more common among caregivers providing more intensive support. Interest in technology for caregiving far exceeded active use, suggesting an opportunity for technology innovation and expansion to better meet the needs of these individuals and their care recipients. Our findings also highlight important information-sharing barriers that can be addressed by health systems to more fully engage out-of-home caregivers in the health care of chronically ill patients.

According to a recent Pew Internet survey, close to 80% of caregivers now have access to the Internet, and approximately two-thirds of online caregivers report that their last Internet health information search was on behalf of another person, suggesting that use of technology to support informal caregiving activities is pervasive [17]. Few studies, however, have investigated technology use and its desirability among caregivers who live apart from their care recipients. A recent National Alliance for Caregiving report revealed that individuals providing care from a distance were more likely than their in-home counterparts to report that technology could make them more effective as caregivers [18]. Our study builds on this report by describing specific technology applications that are used most frequently by out-of-home caregivers, and by identifying barriers to technology use among these individuals.

One technological feature of great interest to out-of-home caregivers in our study (both active technology users and technology nonusers) is the ability to interact with their care recipient’s health care system, for example to communicate with a provider or monitor laboratory results. Many of these out-of-home caregiving technology functions could potentially be performed through a patient portal or electronic personal health record (PHR) [19,20]—tools that are increasingly available through various health care systems, including Kaiser and the Veterans Health Administration. Several studies have documented growing interest in adapting PHRs to enhance information sharing among patients, their caregivers, and their network of health care providers. For example, in a study of more than 18,000 users of the Veterans Affairs’ My Health*e*Vet PHR, approximately 80% expressed interest in sharing access to their record with a family member, caregiver, or provider outside the Veterans Affairs system [21]. Other studies indicate that caregivers are similarly interested in having remote access to their care recipient’s electronic health information [22,23].

Unfortunately, despite patient and caregiver preferences for information sharing, many health care systems impose barriers that limit such communication. In our survey, nearly half of technology-using caregivers (48.6%, 53/109) indicated that health system privacy rules impede their ability to use technology for out-of-home caregiving activities. Patients who wish to share their electronic health information are frequently limited in terms of the specific individuals to whom they may authorize access, and the process is often cumbersome and may require legal documentation [24-26]. Although these regulations stem from reasonable data security considerations, when too restrictive, they may prevent patients from using PHR systems in the ways they find most valuable [27]. Our findings suggest that health systems should consider delegation applications that enable patients to easily share their electronic health information with caregivers.

An additional barrier to technology use for caregiving was unfamiliarity with available programs, despite the fact that Web-based and mobile applications designed specifically for caregivers abound [28,29]. A previous survey of caregivers (both in-home and out-of-home) identified other obstacles to caregivers’ use of technology, including perceived cost (37%) and potential resistance by the care recipient (20%) [18]. These findings suggest that current technologies are either not adequately disseminated to or are not meeting the needs of caregivers and their care recipients, and that the implementation of existing caregiving technology would benefit from a greater user-centered focus.

It should be noted that we used a broad definition of caregiving for this study, including all individuals who engage in at least 1 of 5 common out-of-home caregiving activities. Historically, the term *caregiver* has been used to refer to individuals who provide fairly intense and task-oriented care [9], but there is growing awareness that many caregivers do much more than assist with basic activities of daily living. Caregivers commonly help with chronic illness management tasks, such as medication adherence, tracking of blood pressure or sugar, communication with patients’ health care providers, and health system navigation [30,31]. Because these tasks are not reliant on physical proximity, they may be particularly amenable to support through technology.

### Limitations

Several limitations to our findings warrant discussion. First, although Knowledge Networks maintains a nationally representative panel, the subset of participants who met our criteria might not represent all out-of-home caregivers for adults with chronic illness. In addition, because all of Knowledge Networks’ panelists have Internet access, either independently or as compensation for their panel participation, rates of technology use for caregiving may be higher among survey respondents than in the general population (where 78% have Internet access) [32]. Second, the asynchronous nature of our surveys may have resulted in certain characteristics of survey respondents (eg, caregiving intensity) and care recipients (eg, health status) changing between Wave 1 and Wave 2. Third, we relied exclusively on self-reported data, which could have resulted in recall bias, especially with regards to caregiving intensity and care recipients’ health care utilization. Fourth, our assessment of technology use for out-of-home caregiving may be an underestimate because (1) we queried survey respondents about their use of technology to care for only 1 out-of-home care recipient even if they provide care to multiple individuals, and (2) our survey did not include some emerging caregiving technologies, such as telehealth, videoconferencing, and mobile applications. Finally, this study focused on caregiving for chronically ill adults who are independent in basic activities of daily living; thus, findings cannot be generalized to caregivers of children or individuals with severe cognitive or functional impairments, such as dementia.

### Conclusions

In conclusion, our study suggests that health information technology use is common among out-of-home caregivers for adults with chronic conditions, especially among those providing more intensive care. Both active users and nonusers of technology indicated high levels of interest in expanding their use of technology and adopting new applications for caregiving purposes. The gap between interest and use, as well as barriers cited by survey respondents, should guide technology development and regulations to better address the needs of out-of-home caregivers. Additional investigation is needed to further elucidate specific technology features that are of greatest value to out-of-home caregivers and their care recipients, and to identify the applications that most improve chronic disease management and clinical outcomes. Out-of-home caregivers, armed with remote access to patient health information and their health care team, represent a promising opportunity to enhance chronic disease care, although we need to develop thoughtful implementation procedures and policy to ensure that we achieve this potential.

## Multimedia Appendix 1

Survey about technology use among out-of-home caregivers.

## Multimedia Appendix 2

Full multivariable logistic regression model of caregiver characteristics associated with health information technology use for out-of-home caregiving (N=301).

## Multimedia Appendix 3

Multivariable logistic regression model of care recipient characteristics associated with caregiver’s use of health information technology to facilitate out-of-home caregiving (N=302).

## Abbreviations

CI: confidence interval
OR: odds ratio
PHR: personal health record
