# Supplementary material for: Facilitating Out-of-Home Caregiving Through Health Information Technology: Survey of Informal Caregivers’ Current Practices, Interests, and Perceived Barriers
Source: J Med Internet Res. 2013 Jul 10;15(7):e123. doi: 10.2196/jmir.2472 (PMC3713893; doi:10.2196/jmir.2472)
Supplement: Supplementary file 1 [file jmir_v15i7e123_app1.pdf]

Survey Questions

We are interested in learning about how people use the computer, internet, and e-mail to help friends or relatives outside the home with their health. For example, some people use the computer or internet to track medical information, communicate with health care providers, search for health information or buy prescription medications.

Previously in [XMONTH], 2010, you responded to a survey about how you currently help, or would like to help, your [XRELAT] [XNAME] manage his or her chronic health problems. We would like to ask you some additional questions about your use of the computer and the internet for these activities.

We hope to use this information to design future programs that help people better manage their health conditions together with their family and friends.

[PROMPT]

1. Are you still in contact with your [XRELAT] [XNAME]?
    a. Yes
    b. No

    [*(NOTE: if the respondent answers b, this ends the survey)*]

[PROMPT]

2. Do you currently live with your [XRELAT] [XNAME]?
    a. Yes
    b. No

    [*(NOTE: if the respondent answers a, this ends the survey)*]

[PROMPT]

3.1 In the past year, in what ways have you used the computer, internet, or e-mail to help your [XRELAT] [XNAME] manage his or her health:

a. I helped him or her find health information online

b. I sent messages to his or her doctor or other health care provider by

e-mail

c. I helped him or her track his or her health information (for example their blood pressure, blood sugar, or medication use) on a computer
d. I helped him or her access his or her health records through a system linked to his or her health care provider (i.e., a personal health record system or health portal)
e. I helped him or her use a health portal or personal health record system that is available through his or her health care provider
f. I helped him or her fill medications or medical supplies online
g. I helped him or her look up medical test results online
h. Other ways: ____
i. I have not used the computer, internet, or e-mail for any of the above **[SP]**

[ASK 3.2 IF 3.1=A TO H]

3.2 Over the past year, how frequently have you used a computer or internet to help your [XRELAT] [XNAME] with his or her health in the ways you marked above?

a. Daily
b. 1-5 times per week
c. 1-3 times per month
d. Less than once per month

[ASK Q3.3 IF (Q3.1=I) OR (NO FOR ANY Q3.1 A TO H)]

3.3 **[*For any options in 3.1 for which the respondent answered NO, or for all options in 3.1 if the respondent answered (I):*]** If you thought it would help your [XRELAT] [XNAME] improve their health, and if [XNAME] agreed, how likely would you be to help him or her in the following ways:

a. Help him or her find health information online
b. Send messages to his or her doctor or other health care provider by e-mail
c. Help him or her track his or her health information (blood pressure, blood sugar, or medications) on a computer
d. Help him or her keep track of his or her health records on the computer

e. Help him or her use a health portal or personal health record system that is available through his or her health care provider
f. Help him or her fill medications or medical supplies online
g. Help him or her look up medical test results online

4. Please indicate whether any of the following are reasons that you [if Q3.1=I, use “do not use the computer or internet,”; if Q3.1≠I OR Q3.1=REFUSED, use “do not use the computer or internet more] to help your [XRELAT] [XNAME] with his or her health:
   a. I don’t have enough time
   b. I am not familiar with the programs or websites that would help me do this
   c. I have a health problem or disability that makes it difficult for me to use the computer and/or the internet
   d. The computer and/or internet is too complicated to use it for health or health care issues
   e. I do not trust the internet to provide accurate information about health issues
   f. [XNAME]’s health care provider has privacy rules that would make it hard for me to see his or her personal information
   g. I prefer to deal with health-related activities in person or over the telephone
   h. [XNAME] does not want my help
   i. [XNAME] does not need my help
   j. Other reasons ____ _________________

5. Please respond to the following statements:

a. I feel very comfortable using computers	1	2	3	4	5
b. I feel very comfortable using the internet	1	2	3	4	5
c. I feel very comfortable using e-mail	1	2	3	4	5

d. I feel very comfortable using text messaging	1	2	3	4	5
e. I feel very comfortable learning how to use new programs on the computer or internet	1	2	3	4	5
f. The internet helps me care for my health	1	2	3	4	5
g. The internet helps me manage my health care	1	2	3	4	5

Finally, we have just a few additional questions about your [XRELAT] [XNAME]:

6. How old is [XNAME]? (If you are not sure, please give your best guess)

7. Does [XNAME] use the internet? (Yes/No)
