# Supplementary material for: Facilitating Out-of-Home Caregiving Through Health Information Technology: Survey of Informal Caregivers’ Current Practices, Interests, and Perceived Barriers
Source: J Med Internet Res. 2013 Jul 10;15(7):e123. doi: 10.2196/jmir.2472 (PMC3713893; doi:10.2196/jmir.2472)
Supplement: Supplementary file 2 [file jmir_v15i7e123_app2.pdf]

	n (%)	Unadjusted OR (95% CI)	Adjusted OR (95% CI)	*P* value
Caregiver's age, mean (SD) = 50 (15)		1.00 (0.98, 1.01)	1.01 (0.99, 1.03)	.46
Female	199 (63.0)	0.96 (0.60, 1.55)	1.10 (0.64, 1.90)	.72
Race/Ethnicity				
White, Non-Hispanic	189 (59.8)			
Black, Non-Hispanic	71 (22.5)	1.85 (1.05, 3.25)	1.86 (0.98, 3.53)	.06
Hispanic	56 (17.7)	1.16 (0.62, 2.19)	1.11 (0.55, 2.45)	.78
Education				
Less than high school	29 (9.2)			
High school	72 (22.8)	0.85 (0.33, 2.19)	1.04 (0.36, 2.98)	.94
Some college	112 (35.4)	1.33 (0.56, 3.20)	1.64 (0.60, 4.46)	.34
Bachelor's degree or higher	103 (32.6)	1.30 (0.54, 3.14)	1.43 (0.50, 4.09)	.50
Annual Income				
<$50,000	136 (43.0)			
≥$50,000 and <$100,000	126 (39.9)	0.79 (0.47, 1.33)	0.79 (0.42, 1.46)	.46
≥$100,000	54 (17.1)	1.36 (0.71, 2.59)	1.17 (0.53, 2.56)	.70
Fair/Poor Health Status	142 (44.9)	0.91 (0.70, 1.18)	1.29 (0.66, 2.52)	.46
Out-of-Home Caregiving Intensity				
Very low	125 (39.6)			
Low	104 (32.9)	1.82 (1.03, 3.23)	1.88 (1.01, 3.50)	.05
Moderate	49 (15.5)	2.18 (1.08, 4.41)	2.39 (1.11, 5.15)	.03
High	38 (12.0)	3.91 (1.83, 8.36)	3.70 (1.62, 8.45)	.002
Caregiver's Comfort with Technology				
Very low	46 (14.6)			
Low	42 (13.3)	1.64 (0.61, 4.42)	1.23 (0.41, 3.67)	.72
Moderate	114 (36.1)	2.31 (1.01, 5.26)	2.09 (0.87, 5.02)	.10
High	114 (36.1)	2.88 (1.27, 6.54)	3.49 (1.34, 9.11)	.01

[a] Out-of-home caregiving intensity refers to the number of caregiving activities that the respondent endorsed: assisted care recipient with independent activities of daily living in past three months, assisted care recipient with health-related tasks in past three months, discusses health with care recipient usually or always in conversations, conversed with care recipient's doctors in past year, and advised care recipient about questions to ask health care provider in past year. This measure was scored as very low (1), low (2), moderate (3), and high (4-5).

[b] Caregiver's comfort with technology is measured using the sum of technology modalities or tasks with which the respondent feels comfortable (including computers, the Internet, email, text messaging, and learning new programs on a computer or the Internet). This measure was scored as very low (0), low (1-2), moderate (3-4), and high (5).
