# Supplementary material for: Facilitating Out-of-Home Caregiving Through Health Information Technology: Survey of Informal Caregivers’ Current Practices, Interests, and Perceived Barriers
Source: J Med Internet Res. 2013 Jul 10;15(7):e123. doi: 10.2196/jmir.2472 (PMC3713893; doi:10.2196/jmir.2472)
Supplement: Supplementary file 3 [file jmir_v15i7e123_app3.pdf]

Care recipient characteristics[a]	n (%)	Unadjusted OR (95% CI)	Adjusted OR (95% CI)
Age, mean (SD) = 62 (16)		1.00 (0.98, 1.01)	1.00 (0.98, 1.02)
Internet use	183 (57.9)	1.25 (0.78, 2.00)	1.25 (0.72, 2.16)
Fair/poor health status	142 (44.9)	1.02 (0.79, 1.31)	1.07 (0.65, 1.77)
Difficulty managing health or health care (N = 307)	260 (84.7)	1.51 (0.76, 2.99)	1.49 (0.73, 3.04)
Stayed overnight in hospital in past year	88 (27.9)	1.05 (0.62, 1.75)	1.04 (0.60, 1.79)
Visit to emergency room in past year	76 (24.1)	0.98 (0.57, 1.69)	0.88 (0.50, 1.54)
Distance from caregiver		1.08 (0.93, 1.25)	1.07 (0.92, 1.25)

[a] All care recipient characteristics are caregiver-reported.
